# Multi-omics approach for comparative studies of monoclonal antibody producing CHO cells

**DOI:** 10.1186/1753-6561-9-S9-O8

**Published:** 2015-12-14

**Authors:** Camila A Orellana, Esteban Marcellin, Trent Munro, Peter P Gary, Lars K Nielsen

**Affiliations:** 1 Australian Institute for Bioengineering and Nanotechnology (AIBN), The University of Queensland, Brisbane, QLD 4072, Australia

## Background

Monoclonal antibody (mAb) therapy has revolutionized the treatment of a vast range of diseases, mostly in the areas of oncology and autoimmune/inflammatory disorders[1]. With a world market exceeding 60 billion USD per year and six mAb related products in the top 10 selling drugs, the industry continues to grow at a fast rate[2]. Chinese hamster ovary (CHO) cells are the preferred production host for therapeutic production of mAbs due to their efficiency in performing post-translational modifications and their ability to produce proteins with similar properties to native human proteins[3]. Surprisingly, despite products varying only by a few amino acids in the variable region of a MAb, each production cell line is still developed by generating and screening a large strain pool, and generally the production process has to be reoptimised.

Systems biology can be used as a powerful tool for the identification of key markers of good production lines, with the aim of engineering superior host lines that more reliably produce good production clones. To date systems biology efforts have been hampered by the need to use the mouse, rat and/or human genome as a reference and has suffered from the inherent limitation in coverage of 2-dimensional gel electrophoresis or mouse or CHO cDNA microarrays. The development of new techniques such as RNA sequencing for transcriptome analysis and LC-MS/MS for proteome analysis combined with the recent release of the CHO genome has reignited interest in using quantitative proteomics and transcriptomics to study high productivity cell lines.

## Materials and methods

Here we applied the latest generation of tools to two CHO cell lines that produce different levels of mAb, as described in Orellana et al[4]. The two cell lines were derived from one transfection pool using the same plasmid carrying genes for a monoclonal antibody. For each cell line, three independent vials were thawed and passaged for two weeks prior to bioreactor inoculation. Cells were cultivated in 700 ml EX-CELL® CD CHO Fusion Medium (Sigma Aldrich) containing 25 µM L-Methionine sulfoximine as selection, in a 1L Mini-Bioreactor (Applikon Biotechnologies) operated at 125 rpm stirring speed, 37°C, pH 6.9 and dissolved oxygen at 50% air saturation.

RNA and protein were extracted from cells harvested in mid exponential phase. RNA samples were analysed with RNA sequencing (RNA-Seq) using the Illumina Hiseq2000 platform and 100 bp paired-end reads. TopHat and Cufflinks open-source software[5] were used with default settings for gene expression analysis, using the CHO genome as reference. Protein samples were analysed using SWATH[6]. The Paragon Algorithm from ProteinPilot v4.5 (ABSciex, Forster City CA)[7], PeakView v.1.2 software (ABSciex, Forster City CA) and the R package Limma[8] were used for data analysis. Transcripts and proteins were classified as differentially expressed if the adjusted p-value (Benjamini-Hochberg) was lower than 0.05.Gene set enrichment analysis was performed using DAVID Bioinformatics functional annotation tool[9].

## Results and discussion

The high producer cell line displayed a slightly slower growth rate of 0.0310 ± 0.0002 h-1 compared to the low producer cell line with 0.0340 ± 0.0004 h-1. The two cell lines achieved a titre of 104.3 ± 5.5 mg/L and 52.9 ± 2.0 mg/L on day 4 respectively, with a four-fold difference in mAb specific productivity (19.5 ± 1.0 pg/cell/day compared to 4.6 ± 0.2 pg/cell/day).

**Figure 1:**
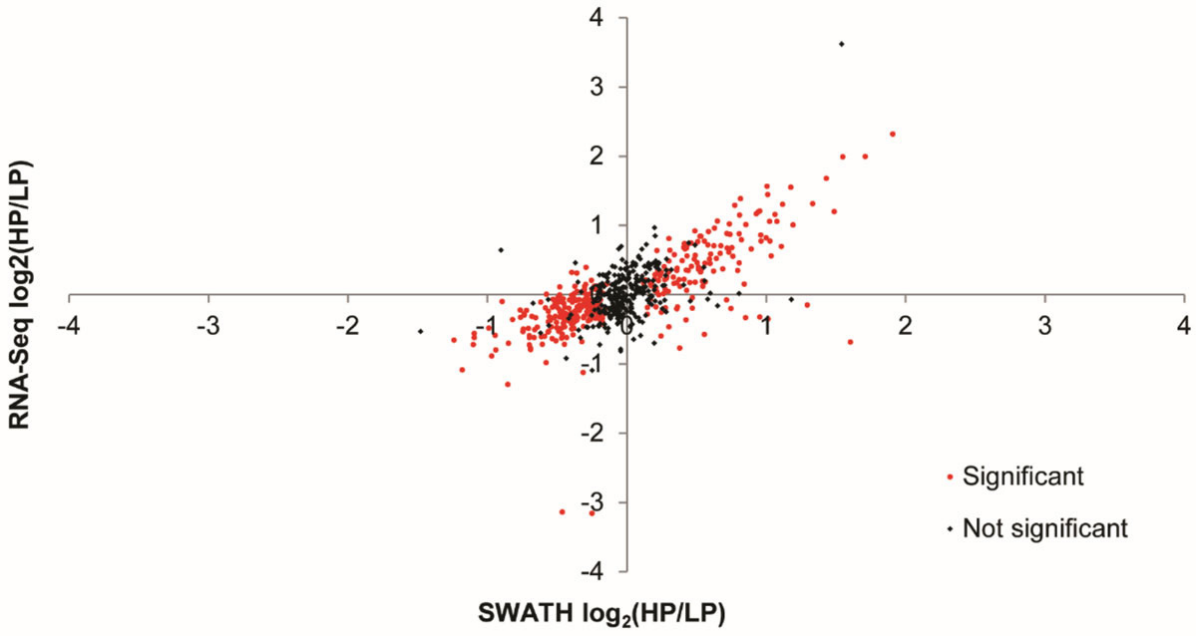
**RNA-Seq and SWATH log_2_-transformed fold change between high producer (HP) and low producer (LP) cell lines of common transcripts/proteins**. Protein with a minimum of 2 peptides with 95% confidence were used. Highlighted in red are the proteins classified as differentially expressed using SWATH technique.

More than 100 million reads were obtained for each sample by RNA-Seq, with more than 83% of the reads mapping to the CHO reference genome.14,300 transcripts and 714proteins were quantified and tested for differential expression. Despite the fact that both clones come from the same transfection pool, 58% of the quantified transcripts and 56% of the quantified proteins varied significantly. Figure 1 shows the log2-transformed fold change between the high producer and the low producer cell lines for common transcripts/proteins between RNA-Seq and SWATH techniques. For most of the genes (80%), the direction of regulation, i.e. up- or down-, agreed between transcripts and proteins.

Three key biological processes were identified by proteomics as up-regulated in the high producer cell line: glutathione biosynthesis, actin filament processes and intracellular transport, while several growth-related processes were down-regulated as expected from the lower growth rate. Metabolomic analysis confirmed that the high producing cell line displayed higher intracellular levels of glutathione. These processes may be important for conferring high mAb production, and as such gene candidates have been nominated for targeted engineering of high-expression cell lines.

## Acknowledgments

The authors would like to thank Robin Palfreyman, the Queensland node of Metabolomics Australia, the proteomics facilities at SCMB, and SAFC - Sigma Aldrich.
